# Enhancing quality of health care delivered to under 5 children in Kibra informal settlement of Nairobi, Kenya, by using mHealth platform with electronic clinical decision support system

**DOI:** 10.1093/oodh/oqaf030

**Published:** 2025-11-14

**Authors:** Afeworki Abraham, Rashed Shah, Barry Finette, Elsie Nzale Sang, Megan McLaughlin, Ezra Mount-Finette, David Oot

**Affiliations:** Department of Health and Nutrition, Save the Children International—Kenya, Benca Court, Muigai Estate, Kitengela, PO Box 19296, Nairobi 00100, Kenya; Department of Global Health, Save the Children US, 899 N. Capitol Street NE, Washington DC 20002, USA; Department of Pediatrics, Microbiology and Molecular Genetics, University of Vermont Larner School of Medicine, 89 Beaumont Ave, Burlington, VT 05405, USA; THINKMD, 50 Lakeside Ave, Burlington, VT 05401, USA; Department of Health and Nutrition, Save the Children International—Kenya, Benca Court, Muigai Estate, Kitengela, PO Box 19296, Nairobi 00100, Kenya; THINKMD, 50 Lakeside Ave, Burlington, VT 05401, USA; THINKMD, 50 Lakeside Ave, Burlington, VT 05401, USA; Department of Global Health, Save the Children US, 899 N. Capitol Street NE, Washington DC 20002, USA

**Keywords:** mHealth, eCDSS, QoC, adherence, IMNCI, HCP, Kenya

## Abstract

This study evaluated an mHealth platform with embedded electronic clinical decision support system, which is compliant with World Health Organization and United Nations International Children’s Emergency Fund recommended Integrated Management of Newborn and Childhood Illnesses (IMNCI) guidelines and designed to support health care providers (HCPs) to assess, diagnose and manage common childhood illnesses in low-resource settings. A quasi-experimental study was conducted between February 2019 and March 2020 in the Kibra informal settlement of Nairobi, Kenya, to evaluate the mHealth platform’s effectiveness in improving the quality of child health services. The intervention included mHealth-supported service delivery, supportive supervision, community outreach, and targeted both public and private healthcare facilities. The primary outcome was increased adherence to IMNCI guidelines by HCPs, measured through comparisons between baseline and endline clinical assessments. Among the 89 HCPs utilizing the mHealth platform, the proportion selecting the World Health Organization recommended amoxicillin dispersible tablets for treating childhood pneumonia increased from 3% at baseline to 38% at endline. Additionally, HCP's awareness regarding the non-indicated use of antibiotics for simple diarrhea management increased from 14% to 50%. Over 90% of HCPs demonstrated compliance with IMNCI protocols at endline, and 85% reported increased clinical confidence attributable to use of the mHealth platform. Following these results, the program was scaled to eight additional sub-counties across Nairobi from 2021 to 2024 when 232 trained HCPs served 26 254 children under 5 years of age, using the mHealth platform. These findings provide evidence to support implementing mHealth platforms with embedded electronic clinical decision support system to enhance IMNCI guideline adherence, increase provider confidence and improve the quality of pediatric care in resource-limited settings.

## Graphical Abstract

**Graphical Abstract:**
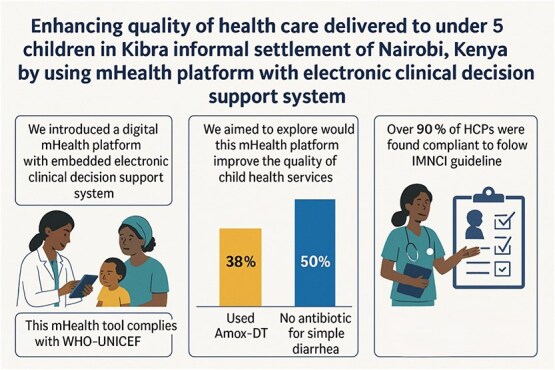

## INTRODUCTION

The Integrated Management of Newborn and Childhood Illness (IMNCI) strategy, developed by the World Health Organization (WHO) and the United Nations International Children’s Emergency Fund (UNICEF), was introduced in the 1990s and has since been adopted by over 100 countries [1]. A 2016 Cochrane review by Gera *et al.* found that IMNCI implementation at scale in health facilities and communities was associated with a 15% reduction in child mortality [2].

Traditionally, IMNCI has been implemented using a paper-based chart booklet, which guides health care providers (HCPs) through step-by-step procedures for classifying, diagnosing and managing childhood illnesses. This chart booklet also functions as a data collection and reporting mechanism. However, its manual nature may hinder workflow efficiency, reduce usability and limit consistent application by HCPs, ultimately serving as a deterrent to their adherence to IMNCI guidelines and affect quality of care [3].

Studies have shown that HCP adherence to IMNCI protocols is often suboptimal across both public and private sectors [4, 5]. Contributing factors include inadequate training, supervision gaps, lack of access to essential medicines and equipment, and health system fragmentation [6–9]. Additionally, HCPs often rely on personal clinical experience rather than standardized guidelines, which can erode the habitual use of IMNCI tools over time [10–13].

Globally, nearly one billion people live in urban slums [14] where the risk of illness and death to children under 5 years of age is often higher than their rural counterparts [15]. Yet, these settings remain underserved by public health infrastructure. Public sector health services in urban areas (like Nairobi), and especially in urban informal settlements (like Kibra), have not kept pace with urban population growth, being neither accessible nor responsive to the critical health needs of the residents in such informal settlement areas [16]. As urban populations continue to grow—virtually all future population growth is projected to occur in urban areas—the demand for accessible, high-quality health services in informal settlements is projected to increase exponentially—and Kibra in Nairobi, Kenya is no exception [17]. Accessibility to and quality of health care services in such informal settlement areas are often significantly affected by many factors, including: geographic distance, overcrowding, long wait times, lacking continuity of care and insufficient regulation of private providers [18]. Although the Kenyan government has made efforts to expand healthcare access in underserved urban areas, public health facilities in Kibra remain limited in number and are often overstretched by the high population density.

Results from a study conducted in Kenya in December 2016 [19] revealed that women and children in Kibra settlement initially seek health care from private clinics or chemist shops. Findings from this cross-sectional health care seeking assessment also documented that majority of the health practitioners often delivered care that was substandard, or in some cases, harmful. Kenya’s healthcare system is organized into six levels, ranging from community health units to national referral hospitals, each serving distinct roles in providing healthcare services. The Ministry of Health (MoH) run public facilities enrolled in the study were either dispensaries or health centers, which are level 2 or level 3 facilities within the Kenyan health care system, respectively. Services offered by dispensaries in cities in Kenya are similar like those provided by health centers, with the difference that the dispensary does not have in-patient facilities. Additionally, health centers offer specialized clinics for the management of non-communicable diseases (e.g. Diabetes, TB etc.). Both dispensaries and health centers are run by either nursing officers or clinical officers. Doctors may also be available to provide services in health centers. Given the context of health care delivery system in Kibra informal settlement, after we shared these results with the MoH in Kenya, and specifically with the health management teams at the county and sub-county level, they suggested to implement an mHealth platform compliant with WHO’s recommended IMNCI guidelines. Mobile health (mHealth) refers to an area of electronic health (eHealth) where the use of mobile technologies such as smartphones, tablets and personal digital assistants (PDAs) to deliver health services and/or relevant information [20]. Researchers reported that mHealth improves the ability of HCPs to accurately diagnose, treat and refer, when necessary, for common childhood illnesses in resource-limited settings. The mHealth tools can help enhance efficiency, improve health worker decision-making, and quality of care and health outcomes. Context of digitalized data and increases access to modern healthcare in rural areas of LMIC [21–24].

Specifically, two meta-analyses found that the electronic clinical decision support system (eCDSS) tools improved health worker quality of care [25, 26],, and especially their compliance with IMNCI guidelines when compared to the paper version of IMNCI [12, 13, 27]. The eCDSS tool has the potential to enable basic community-level HCPs, including HCPs from NGO/FBO, drug dispensers/pharmacists at chemist shop) to correctly follow relatively complex charts, such as the IMNCI Chart Booklet [28].

### Rationale

Despite the widespread adoption of IMNCI, adherence by HCPs remains suboptimal, particularly in urban informal settlements with limited public infrastructure and oversight of private providers. There is limited evidence on the effectiveness of digital IMNCI-compliant mHealth platforms in improving provider adherence and quality of pediatric care in these settings. This study addresses this gap by evaluating whether an mHealth platform with embedded eCDSS improves clinical performance and IMNCI adherence among HCPs in Nairobi’s Kibra informal settlement.

### Research hypothesis and objective

In this study, an mHealth platform was deployed to support frontline health workers with a digital medical intelligence platform with embedded eCDSS that enhance clinical decision-making and adherence to evidence-based care guidelines. We hypothesized that the mHealth platform used in this study would enable health providers to apply an eCDSS to better assess, classify, diagnose and manage common childhood illnesses (e.g. diarrhea, pneumonia and malaria). The THINKMD team designed and developed this WHO-compliant mHealth platform embedded with an eCDSS which operationalizes IMNCI guidelines in a digital format, facilitating systematic patient assessment, diagnosis and treatment decision-making by HCPs. (THINKMD is a social impact benefit corporation, and healthcare technology company which is affiliated to University of Vermont, USA. THINKMD has built the mobile clinical platform (known as MEDSINC) that gives non-healthcare professionals the ability to diagnose and treat the health of newborns, children under five and pregnant and new mothers in any setting. The platform uses evidence-based medicine and Bayesian cluster-pattern recognition logic to guide non-medical professionals in the assessment of a patient using the same logic and approach as physicians. This is usable as eCDSS at point-of-care with the assessment, triage, counseling, and referral capability for children under 5 (including newborns) and pregnant and new mothers (i.e. ante- and postnatal care).)

The objective of this study was to evaluate whether an mHealth platform with embedded eCDSS, supported by supervision and community outreach, could improve HCPs’ knowledge, skills and adherence to IMNCI protocols when assessing and managing sick children under five in Kibra informal settlement in Kenya, over a 12-month period.

## KEY INTERVENTIONS

### MoH partnership and engagement

Engagement with MoH counterparts commenced at the planning stage and was sustained throughout implementation. MoH officials participated in the baseline assessment, guided the contextual adaptation of the eCDSS and mHealth platform and oversaw the selection and training of master trainers and HCPs. In addition, MoH personnel were involved in field-level monitoring, supportive supervision visits and coordination of both baseline and endline evaluations.

This sustained and systematic collaboration was instrumental in fostering government ownership of the intervention. It also ensured that implementation was responsive to the local health system context and supported the longer-term goal of integrating digital clinical tools into routine service delivery. Importantly, this engagement laid the groundwork for continued MoH commitment to quality improvement initiatives beyond the pilot phase.

### Implementation of mHealth platform with embedded eCDSS

**Figure 1:**
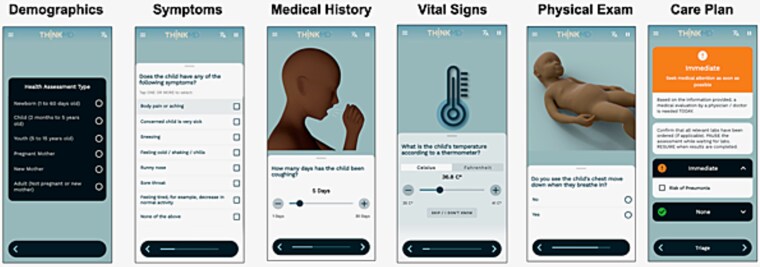
Clinical decision support platform panels (courtesy: THINKMD).

The mHealth platform with embedded eCDSS had previously been validated in multiple low- and middle-income country settings and is specifically designed to strengthen care quality in resource-constrained environments [29]. It captures 42 key clinical data elements derived from IMNCI and Integrated Community Case Management protocols, as well as additional evidence-based criteria. It employs a physician-informed Bayesian logic engine to synthesize the collected data and generate clinical risk assessments, severity scores, triage recommendations and treatment options [29–31]. Figure 1 illustrates the platform workflow and decision architecture.

### Technology provision and technical support

To operationalize the mHealth intervention, handheld mobile devices (e.g. tablets) were procured and preloaded with the THINKMD platform. These devices were distributed to trained HCPs for use during clinical encounters with children under 5 years of age at designated service delivery points. To ensure continuous functionality and uptake of the digital tool, a system for on-demand troubleshooting and technical support was established. This support structure enabled rapid resolution of software, device and user-related issues, ensuring that technology access did not disrupt clinical workflow.

### Monitoring and supportive supervision

Save the Children and MoH team jointly conducted on-site monitoring and supportive supervision visits to participant HCPs which served multiple purposes: reinforcing consistent use of the eCDSS tool, addressing user feedback, resolving content and functionality-related questions, and reviewing performance and service delivery data generated by the platform. These supervisory engagements helped sustain provider motivation and ensured fidelity to the intervention.

### Community engagement and care-seeking promotion

To improve timely care-seeking behavior among caregivers and enhance early recognition of childhood illness, community outreach was integrated into the study. A total of six community health assistants and 88 community health volunteers (CHVs) received a 1-day orientation focused on educating caregivers about identifying danger signs, the importance of timely care-seeking, and how to access services from THINKMD-trained providers within their communities. This community-facing component aimed to strengthen the continuum of care by ensuring that families were informed, engaged and connected to high-quality service delivery points.

#### Training

A cascaded training model was used to scale implementation of the digital platform. Under the leadership of the MoH and Save the Children, with technical input from THINKMD, 15 Master Trainers were selected in consultation with the sub-county health management team. These master trainers subsequently conducted multiple rounds of training for frontline HCPs.

Each training cohort consisted of 16–20 HCPs and included the following components:

- overview lecture introducing IMNCI principles and the mHealth tool
- demonstration sessions of the digital platform and clinical logic
- interactive role play and structured case study discussions, and
- hands-on practice using the tool during supervised live patient encounters.

## METHODOLOGY

The study employed a quasi-experimental pre-post design (without a formal control group) and was conducted in Kibra, an expansive informal settlement located on the outskirts of Nairobi, Kenya, between 1 May 2019 and 1 April 2020. Kibra (within the Langata administrative region) informal settlement area was selected for this study based on following criteria:

- high population density and child disease burden
- limited public-sector pediatric coverage
- high reliance on informal and private HCPs, and
- supportive engagement from local health authorities.

### Setting

Frequently described as one of the largest urban settlements in Africa, Kibra is home to an estimated 185 777 residents living within a 12.1 km^2^ area [32]. The population endures severe socio-economic deprivation, with the majority of households subsisting on less than USD $2 per day. Residents experience limited access to essential services, including clean water, sanitation, electricity and healthcare infrastructure [33].

Housing in Kibra is typically overcrowded, with multiple family members confined to small, poorly ventilated spaces. Sanitation facilities are inadequate, contributing to heightened exposure to communicable diseases, particularly among children. Although child-specific mortality data for Kibra are unavailable, the 2022 Kenya Demographic and Health Survey reported an under-five mortality rate of 44 deaths per 1000 live births in Nairobi County—exceeding the national average of 41 per 1000 live births [34].

In Kenya, the leading causes of death among children under five remain pneumonia and diarrhea, both of which are preventable and treatable with timely and appropriate care. These conditions are especially prevalent in informal settlements like Kibra, where structural barriers limit early detection, diagnosis and treatment.

### Baseline/endline data capture and analysis

The study had a baseline assessment in February 2019 and an endline assessment in March 2020 and measured the changes by comparing baseline-endline data. Specifically, the study sought to assess the quantitative changes in the primary outcome measures, focused on HCP’s knowledge and practice in assessing, classifying, diagnosing and managing the sick children they encountered, and their adherence to the IMNCI guidelines. The HCPs in this study included qualified medical doctors, nursing officers or clinical officers recognized by the MoH irrespective of their place of work that could be a non-government organization (NGO) or faith-based organization (FBO) run clinic, private clinic, dispensary, health center, MoH run (public) health clinic/center and/or hospital. Occasionally, in the unregulated setting of informal settlements pharmacists and pharmacy technicians conduct clinical work and assess and manage sick children from dispensaries. That is why we also included them in the study. All these participating HCPs were full-time employees at their assigned health care facility.

**Data collection methods and tools** during these assessments included:

- *Structured interviews with HCPs:* Trained research staff conducted face-to-face structured interviews with participating HCPs to assess their knowledge of appropriate diagnosis and treatment for childhood illnesses. Interview content was pre-tested and focused on key IMNCI-related practices, including illness recognition, clinical decision-making and treatment planning.
- *Practice and adherence to IMNCI using observation checklist during on-site observation:* Using an IMNCI observation checklist created by the research team after consulting with MoH and capturing 16 IMNCI data points, derived from WHO IMNCI guidelines, selected based on importance and ease of observational acquisition (i.e. easily heard or seen performed by another person). Each study participant HCP was observed and evaluated by a trained observer who used this standardized checklist while the HCP was assessing encountered children. The trained observer noted, on the checklist, what they observed and heard while the HCP completed their assessment of each child. The same checklist and methodology were used for both baseline and endline assessments.
- *Usability and acceptability survey:* As part of the endline assessment, a structured survey was administered to assess the HCPs’ perceptions of the mHealth platform and eCDSS. The survey captured user insights related to system usability, perceived benefits, integration into workflow and overall satisfaction with the digital tool.

### Data analyses

Data analyses were done using descriptive statistics, frequency distributions and pre-post comparisons of baseline, endline data using standard statistical tests (specifically paired *t-*test, chi-square test, two sample proportion test), as applicable. The study measured quality of care in terms of HCPs’ improved knowledge (e.g. correct identification of danger signs) and better practice (e.g. appropriately prescribing antibiotics for managing childhood pneumonia and/or prescribing a combination of oral rehydration salts (ORS), Zinc and Vitamin A for managing diarrheal illnesses), that adhere with IMNCI guidelines assessment and clinical management as well as care plan decision-making for a sick child.

**Data quality assurance** process included:

- standardized training of observers and interviewers
- pre-testing of structured tools
- regular supervisory checks and double data entry verification, and
- use of platform-generated logs for consistency checks.

### Ethical approval

The study protocol received ethical approval from the following institutions:

- Ethical Review Committee of Save the Children US (Ref #: FWA#00022738; dated 18 October 2018)
- Kenyatta National Hospital—University of Nairobi Ethics and Research Committee (Ref #: KNH-ERC/A/9; dated 11 January 2019)
- Committees on Human Subjects Serving the University of Vermont, USA and the UVM Medical Center (Ref #: CHRMS 15-037; dated 17 September 2018)

## RESULTS

### Study participants and facilities

A total of 89 trained HCPs implemented the mHealth platform across 49 health facilities during the study period. The average age of these 89 participating HCPs was 35 years of age, including 48% male and 52% female. On average, these HCPs had over 10 years of work experience and notably, 65% of them had previously been trained on using the IMNCI protocol.

A breakdown of the participating HCPs by professional category and their distribution at different types of facilities are also shown in Table 1.

**Table 1:** Professional categories of participant HCPs and their distribution at different types of facilities.

Category of HCPs (*N* = 89)	Number of specific categories of HCP (%)	Types of facilities (*N* = 49)	Number of HCPs at specific facility (%)
Medical doctor	01 (1.12%)	Private clinic (13)	19 (21.35%)
Clinical officer	44 (49.44%)	Chemist shop/drug store (11)	18 (20.22%)
Nursing officer	41 (46.07%)	NGO/FBO clinic (9)	21 (23.60%)
Pharmacists/pharmacy technologist	03 (3.37%)	Public (government) health facility (16)	31 (34.83%)

### Evaluation of changes in HCP’s knowledge, clinical practice and compliance with IMNCI guidelines

The evaluation, by comparing base-line and endline data, measured the changes in HCPs’ knowledge and clinical capacity related to their assessment, classification, diagnosis and management of childhood illnesses, as well as their adherence to WHO-recommended IMNCI guidelines during patient encounters.

One key indicator of clinical knowledge was the correct identification of the three primary IMNCI danger signs: inability to drink or breastfeed, lethargy or unconsciousness and vomiting everything. At baseline, only 9% of HCPs correctly identified all three signs. By the endline assessment, this proportion had increased to 44%.

**Figure 2:**
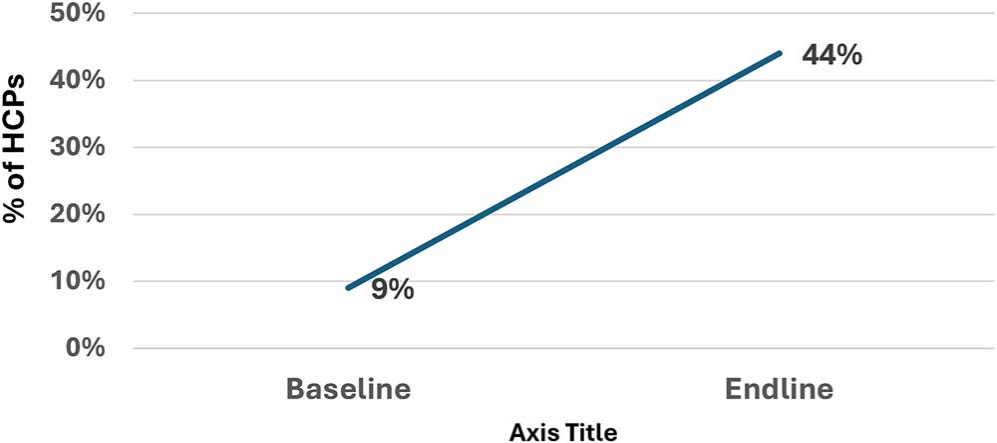
Percentage of HCPs correctly identifying IMNCI danger signs at baseline and endline.

Figure 2 displays the comparative results between baseline and endline assessments regarding HCPs’ ability to identify these danger signs.

In their clinical practice for pneumonia management, the proportion of HCPs who correctly prescribed amoxicillin dispersible tablets (DT) - the antibiotic recommended by WHO for childhood pneumonia - increased markedly from 3% at baseline to 38% at endline.

**Figure 3:**
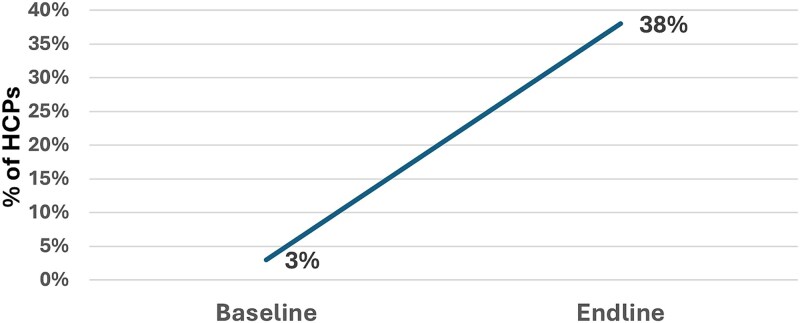
Proportion of HCPs prescribing amoxicillin DT for childhood pneumonia at baseline and endline.

Figure 3 illustrates the change in appropriate antibiotic prescribing for childhood pneumonia between baseline and endline.

In the case of diarrhea management, clinical practice of HCPs following the IMNCI guideline was also improved. At baseline, only 14% of HCPs correctly recommended the combination of ORS, zinc supplementation and vitamin A, as per IMNCI standards. By the endline, this proportion had increased to 50%, reflecting improved clinical knowledge and practice.

Additionally, awareness among HCPs that antibiotics are not indicated for simple diarrhea rose significantly from 14% to 50%.

Table 2 summarizes changes in HCPs’ knowledge and practices related to the management of childhood diarrhea.

**Table 2:** Changes in knowledge and clinical practice of HCPs for management of simple diarrhea.

Knowledge about how to manage simple diarrhea?	Baseline (February 2019)	Endline (March 2020)
Knew to treat with ORS, zinc and vitamin A	58%	71%
Knew not to dispense antibiotics	14%	50%

The proportion of HCPs correctly identifying all three IMNCI danger signs increased from 9% at baseline to 44% at endline (absolute increase of 35 percentage points, *P* < 0.001). Appropriately prescribing amoxicillin DT for management of childhood pneumonia also rose from 3% to 38% (absolute increase of 35 percentage points, *P* < 0.001). Recognition that antibiotics are not indicated for simple diarrhea increased from 14% to 50% (absolute increase of 36 percentage points, *P* < 0.001). Knowledge of using ORS, Zinc and Vitamin A combinedly for managing simple diarrhea increased from 58% to 71% (absolute increase of 13 percentage points) revealed a positive trend although did not reach the significance level with conservative testing (*P* = 0.082).

### HCP’s adherence to IMNCI guidelines

Healthcare providers’ adherence to IMNCI protocols was assessed through direct observation of clinical encounters involving sick children under 5 years of age.

As summarized in Table 3, baseline observations revealed variability in adherence to IMNCI components. The most consistently recorded data points were temperature and presence of cough, while several critical assessments were frequently missed. These included evaluation of pitting edema (24%), documentation of convulsion history (28%), performance of ear examinations (30%), respiratory rate counts (36%) and inquiry about blood in the stool (36%).

**Table 3:** Comparison of baseline and endline observations across 16 key IMNCI clinical assessment indicators.

Observation on selected indicators	Baseline (%)	Endline (%)
Skin turgor	40	84
Examined neck	38	60
H/O convulsion	28	64
H/O blood in stool	36	60
Frequency of stool	44	92
Cough	74	96
Temperature	76	100
Breathing rate	36	92
Exam for rashes	40	76
Eye exam	56	92
Ear exam	30	52
Mouth exam	46	64
Feeding	62	80
Vomiting	52	88
Weight	64	100
Pitting edema	24	80

By the endline assessment, improvements were observed across multiple indicators. Notably, there was a 56 percentage-point increase in the frequency of assessments for both pitting edema and breathing rate, reflecting strengthened clinical examination practices. Additionally, weight and temperature were captured in 100% of observed encounters, indicating full compliance with basic IMNCI requirements for these parameters.

Significant improvements were observed for the acquisition of several key IMNCI clinical assessment indicators. Examples include breathing rate recorded (36% → 92%, *P* < 0.001), pitting edema checked (24% → 80%, *P* < 0.001), temperature measured (76% → 100%, *P* < 0.001), and eye examination (56% → 92%, *P* < 0.001). These findings support a broad-based strengthening of clinical healthcare capacity, quality and assessment fidelity aligned with IMNCI.

### Facility-based trends in IMNCI guideline adherence

**Figure 4:**
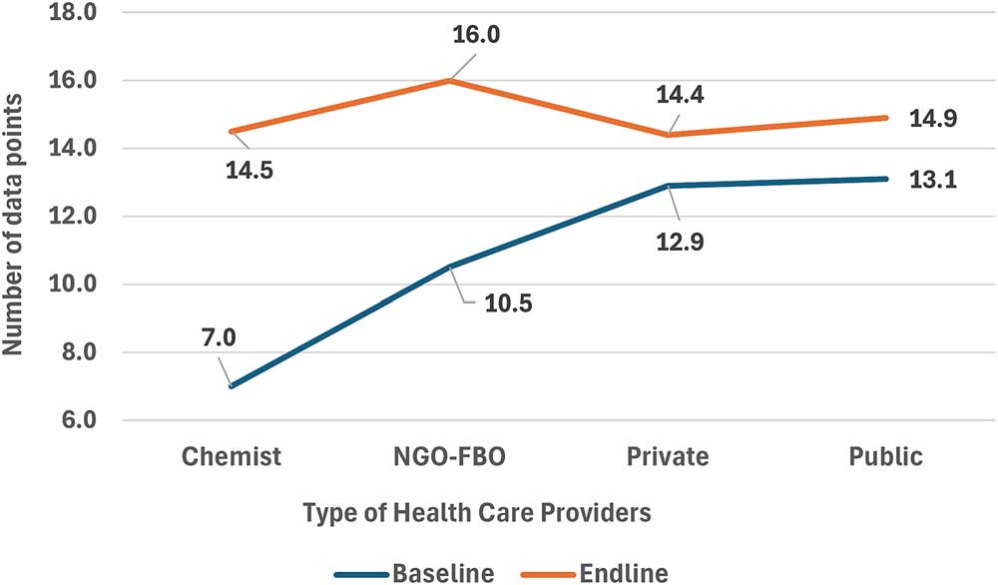
Changes in overall IMNCI guideline adherence among HCPs by facility type, from baseline to endline.

A comparative analysis of baseline and endline observations was conducted to assess changes in overall adherence to IMNCI guidelines among HCPs across different facility types. As shown in Fig. 4, at baseline, HCPs at public sector facilities demonstrated the highest adherence, with an average score of 13 out of 16 IMNCI data points completed per clinical encounter. In contrast, providers at chemist or drug shops recorded the lowest baseline adherence, averaging 7 out of 16 points.

By the endline assessment, improvements were observed across all facility types. Notably, HCPs at chemist/drug shops exhibited the greatest overall improvement, increasing their adherence by 7.5 points.

### Use of mHealth platform by HCPs

During the study period, the platform was used to evaluate 4400 children (as shown in Table 4) under the age of five, including newborns, as part of routine clinical care delivered by the participating HCPs.

**Table 4:** Number of children evaluated by mHealth tool users at different types of facilities.

Type of facility	Number of children evaluated
Public health facility	1951
Private clinic	1608
NGO clinic	355
FBO clinic	223
Chemist shop/drug store	263

On average, the HCPs operating at chemist shops or drug stores completed the fewest child health assessments per day, averaging two encounters daily. In contrast, nurses based at public health facilities recorded the highest average, completing approximately 40 child health encounters per day. Most of the children being seen were infants (0–12 months), and the distribution of female to male children encounters was 49% and 51%, respectively.

**Figure 5:**
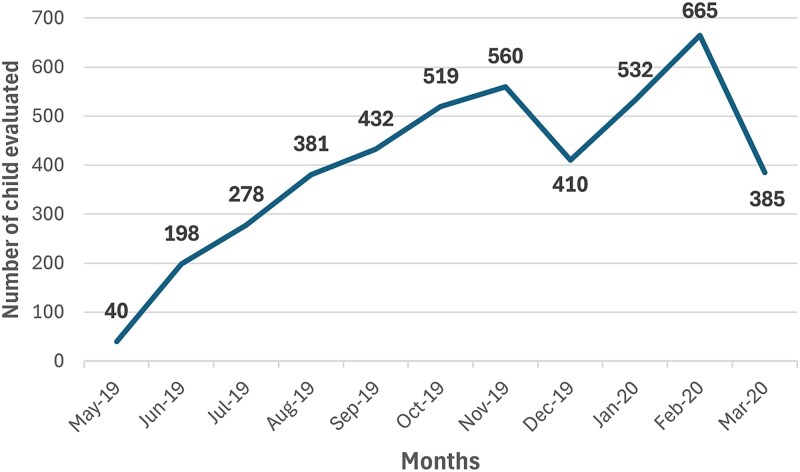
Total number of clinical risk assessments completed over time.

Figure 5 illustrates the temporal trends in mHealth platform utilization across the study period. A notable decline in usage was observed during the November–December 2019 holiday season, when many local residents temporarily relocated to their rural homes. A second significant drop in utilization occurred in March 2020, coinciding with the onset of the COVID-19 pandemic and related service disruptions.

### Usability and acceptability of the mHealth platform

As part of the endline assessment, a structured survey was administered to 89 HCPs who used the mHealth platform during the study period. The survey aimed to evaluate user perceptions regarding the usability, acceptability and perceived value of the digital tool in routine clinical practice.

The majority of respondents, [66 HCPs (74%)], reported that the platform was either very easy or easy to use during patient encounters involving sick children at the point of care. Additionally, 83 HCPs (93%) indicated that they were likely or extremely likely to recommend the platform to colleagues, resulting in a Net Promoter Score (NPS) of 98, with no detractors. (NPS measures how likely a person will recommend a product to another person. It is the single most important question to assess if a product is viable and will have successful adoption. An NPS of >50 is considered very high.) 

Furthermore, 79 HCPs (89%) expressed a willingness to continue using the platform in future clinical practice, reflecting strong acceptability. Importantly, 76 HCPs (85%) reported that use of the mHealth platform enhanced their confidence in making clinical decisions and delivering care.

## DISCUSSION

This study demonstrates that implementation of a WHO IMNCI-compliant mHealth platform with an embedded eCDSS resulted in measurable improvements in HCP knowledge, clinical practice and adherence to WHO guidelines for managing childhood illnesses. Specifically, the intervention led to: (i) improved recognition of key IMNCI danger signs: study result shown in Fig. 2 revealed a marked improvement in critical clinical recognition skills; (ii) enhanced quality of diarrhea and pneumonia management: Results shown in Fig. 3 and Table 2 also highlighted increased understanding and rational practice of prescribing antibiotics for management of childhood diarrhea and pneumonia; (iii) increased prescription of amoxicillin DT, the recommended treatment for childhood pneumonia: Prior to the study, many HCPs tended to prescribe more costly, broad-spectrum, third-generation antibiotics that are not recommended as first-line treatment for uncomplicated pneumonia in children; and (iv) greater overall compliance with IMNCI protocols at the point of care: Data presented in Table 3 suggest that the mHealth platform, coupled with supervision and training, contributed to marked improvements in HCPs’ compliance with IMNCI clinical assessment protocols. Data in Fig. 4 are showing overall adherence to IMNCI guidelines among HCPs across different facility types and their clinical practices in settings that typically operate outside formal regulatory and training frameworks.

Results from this study indicate that the implementation of the mHealth platform—combined with training and supervision—contributed to measurable improvements in IMNCI adherence across all health service delivery settings.

These findings align with previous studies documenting the benefits of eCDSS on provider performance. Other study results from Burkina Faso [28], Tanzania [12, 35], Afghanistan [36] also documented improved knowledge and ability of HCPs who used eCDSS to correctly assess, classify, diagnosis and manage sick children. Improved adherence to the IMNCI protocol by HCPs while they use eIMNCI or eCDSS on mHealth platform are also well documented [12, 28, 31, 35, 37–39]. The mHealth platform used in this study enforced a structured workflow that prevented user HCPs from bypassing critical IMNCI steps, ensuring consistent adherence to clinical standards.

Moreover, results from this study are consistent to other studies suggesting that similar mHealth tool implementing eIMNCI was well received, was easy to use and perceived by HCPs as a useful tool to improve their confidence as well as care of sick children [23, 27, 40, 41]. While initial resistance was noted, some HCPs viewed the tool as time-consuming; standardized, structured workflow efficiency improved significantly with experience. Early use required approximately 12–15 minutes per patient, which decreased to 7–9 minutes as HCPs became proficient. Additionally, findings of this study underscore the platform’s user-centered design, operational feasibility and potential for broader integration within frontline health systems.

Notably, an initial tendency among some providers to prescribe unnecessary or costlier antibiotics due to financial incentives was an important observation. Using the eCDSS mitigated this behavior by guiding HCPs toward evidence-based prescribing practices. Supportive supervision and ongoing counseling further encouraged appropriate antibiotic use, findings echoed in a USAID-commissioned report showing similar improvements in pharmaceutical stewardship through supportive oversight [42]. The improved use of amoxicillin DT, and reduced use of inappropriate more expensive antibiotics should lead to improved clinical outcomes. Future studies will need to investigate clinical endpoints such as morbidity or recovery rates to fully demonstrate the effects of this important behavioral change.

Researchers reported [37] on advantages of using an mHealth platform on a handheld device (either on tablet or phone) which offers a potential opportunity to deliver other services or support, such as distant learning, supply chain management and telemedicine. The use of IMNCI clinical platform with an embedded eCDSS could play a pivotal role in future efforts to improve the quality of the assessment of a child’s health as well as reduce antibiotic overprescribing—a large and growing public health problem [43]. This is particularly true for resource-limited settings where clinical risk assessments of patients can be challenging and pharmaceutical dispensing guidelines are often not followed [44], resulting in devastating effects of rapidly expanding antibiotic resistance in these contexts [45–48].

Additionally, THINKMD mHealth platform’s offline functionality ensured uninterrupted use even in areas with limited connectivity. Automated data synchronization enabled seamless integration with supervisory dashboards, empowering health managers to conduct real-time performance monitoring and adjust priorities based on actionable data.

Concerns about digital tools disrupting patient–provider interactions were not borne out. Instead, caregivers reported higher trust in care quality when the tool was used. Some mothers explicitly stated that the mHealth tool influenced their decision to seek care—a qualitative outcome that suggests such tools may help drive behavioral changes to seek services (see the supplementary material).

Our study results revealed statistically significant gains in provider knowledge, increased healthcare capacity and IMNCI-aligned practice, including identification of danger signs and evidence-based antibiotic use, and thereby support a critical link between the intervention and improved quality of care, acknowledging the quasi-experimental design of the study. These results provide strong evidence that the mHealth platform with embedded eCDSS, implemented with training and supervision, improved quality of care. Such gain in HCPs’ adherence across the full IMNCI assessment further indicates that the tool helped standardize clinical workflows, clinical data acquisition and prevented omission of critical steps.

However, while the mHealth platform, and the use of eCDSS, helped ensuring HCP’s adherence to IMNCI guidelines, achieving its full potential may be limited due to limitations of the primary health delivery centers themselves with respect to the shortage of staff, drugs and equipment (specifically in MoH run facilities), the lack of any systematic MoH oversight of private sector health care services in urban settings (i.e. health care services offered at private clinic, NGO or FBO run clinic), and the prevailing weak referral system. As others have noted [39], IMNCI-based mHealth tools will be most effective when implemented within functional primary healthcare systems.

### Expansion of implementation in small scale and data interoperability

Following the initial study period (2019–2020), the intervention was expanded at the request of the Nairobi County MoH. Between 2021 and 2024 while it was scaled to eight additional sub-counties (Additional eight sub-counties are Embakasi West, Embakasi Central, Embakasi South, Embakasi East, Ruaraka, Roysambu, Mathare and Starehe sub-counties.), reaching 28 254 children under five through 232 trained HCPs across public, private and NGO facilities. During this phase, successful integration of the THINKMD platform into the Kenya Health Information System (KHIS) enhanced data visualization and use of analytics by sub-county health management teams for real-time performance monitoring.

Community engagement through CHVs remained central to encouraging care-seeking. While some HCPs initially perceived the tool as time-intensive, user feedback and community acceptance helped normalize its use over time.

### Limitations

This study’s observational design introduces several limitations. The presence of observers may have influenced provider behavior (i.e. the Hawthorne effect), which may not reflect true performance in unobserved conditions. While real-time data use offers an additional verification layer, future studies should include unannounced supervisory visits to assess independent compliance. Another limitation is posed by our pre-post design which limits causal inference, but the magnitude and consistency of effect across independent indicators are indicative of attribution to the intervention.

The IMNCI data points included in the observation checklist were selected for feasibility of direct observation, which may have introduced observation bias. This study attempted to minimize this through passive observation protocols. Additionally, recall bias may have affected responses during interviews, and actual adherence to treatment by caregivers at home was not measured.

Geographically, the study was limited to the Kibra/Langata sub-county, which may constrain the generalizability of findings. However, Kibra shares characteristics with many informal settlements in Nairobi and/or other LMIC urban contexts.

During the study, there were several challenging contexts and factors those might have affected study results. The emergence of COVID-19 impacted Kibra residents’ life and work. It was not completely known what impact COVID-19 had on the HCPs, but it is likely that the loss of disposable income of Kibra residents has affected their demand for services, including those HCPs who are participating in the pilot program. Another challenge came from the temptation among HCPs to dispense revenue-enhancing drugs. Despite the IMNCI treatment guidelines incorporated into THINKMD, there remains a powerful financial influence on HCPs to dispense more expensive treatments for common illnesses, and to over-treat by dispensing drugs that are not needed. Some technical Issues (for example, occasional issues included battery failure and syncing delays) sometimes affected continued use of the platform by HCPs. Locally arranged technical support helped mitigate these challenges soon after being notified to the study team members.

Ideally, it would have added value to explore cost-effectiveness and/or cost-efficiency of using the mHealth platform. While the study demonstrated strong improvements in care quality, it did not include cost-effectiveness or cost-efficiency analysis, an important consideration for future scale-up assessments.

Finally, additional data regarding adherence to the recommended treatment by caretaker(s) at home (e.g. complying with medication regimens, recommended practices, etc.) and the outcome of treated children would have added value to these study results.

## CONCLUSION

This study describes the use of an mHealth eCDSS platform as a point-of-care decision support system used by HCPs during patient assessments. Its purpose was to ensure standardized, guideline-compliant diagnosis and treatment for common childhood illnesses. The study results demonstrate that a WHO–UNICEF IMNCI-aligned mHealth platform with embedded eCDSS can significantly improve frontline HCPs’ knowledge, clinical performance and adherence to IMNCI guidelines. These improvements represent critical steps toward strengthening the quality of pediatric care in resource-constrained, urban informal settlements. This study also provides evidence to support the scalability of THINKMD and other digital mHealth platforms. In particular, the benefit to be able to import key data into KHIS, the supportive supervision model, and the inclusion of private-sector providers as unique contributions with broader implications for digital health scale-up in LMICs. Moreover, its use may have potential in both private and public sector settings to strengthen clinical quality where supervisory structures are traditionally weaker.

Given the significant role private practitioners play in healthcare delivery in such settings, targeted digital health interventions like this one offer an opportunity to improve service quality at scale. While this study established feasibility, future research, particularly randomized controlled trials, would be valuable in assessing the long-term sustainability and cost-effectiveness of such interventions.

## Supplementary Material

Supplementary_file_oqaf030

## Data Availability

The data underlying this article are available in the main article and in its online supplementary material. Additional data may also be available from the corresponding author on reasonable request.
