# Supplementary material for: Enhancing quality of health care delivered to under 5 children in Kibra informal settlement of Nairobi, Kenya, by using mHealth platform with electronic clinical decision support system
Source: Oxf Open Digit Health. 2025 Nov 14;3:oqaf030. doi: 10.1093/oodh/oqaf030 (PMC12698342; doi:10.1093/oodh/oqaf030)
Supplement: Supplementary_file_oqaf030 [file supplementary_file_oqaf030.pdf]

Supplementary Material: Client's Satisfaction Note

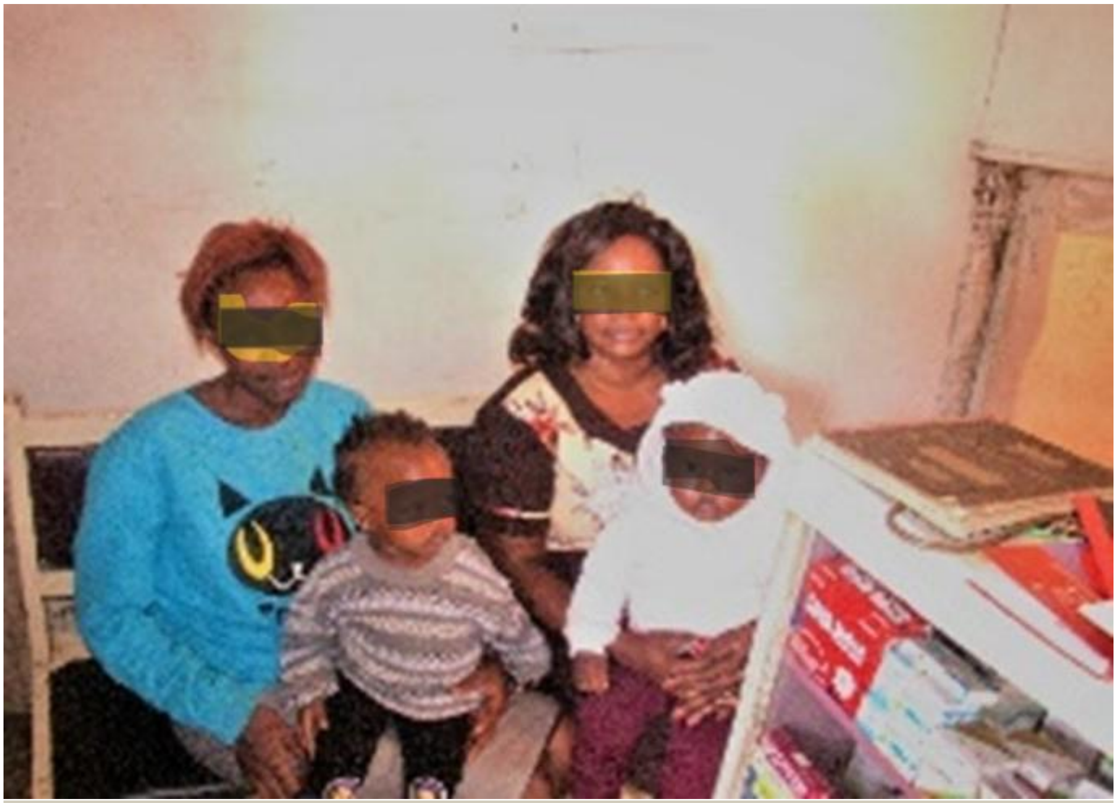

A mother brought her twin daughters to a local Chemist clinic in Kibra as one baby girl developed a cough, diarrhea and a runny nose, while the other one had fever and trouble breathing.

A clinician (on right) examined the girls using the mHealth tool, which she says has improved her clinical skills and led to increased patient satisfaction—including more referrals.

The mother was pleased with the care received: "Previously when I took the girls to a health facility, the nurse would just look at them, ask a few questions and prescribe medicine," she says. "This time, it was different. She performed a very thorough examination, and I had confidence that the medicine prescribed would help. And it did. My girls are doing very well."

Photo: by Save the Children International - Kenya
